# Conceptualising doctors’ and nurses’ experience of formal and informal solidarity: A Meta-Ethnography Protocol

**DOI:** 10.12688/hrbopenres.14294.1

**Published:** 2025-12-05

**Authors:** Maja Dumana, Seán Ó Riain, John Paul Byrne

**Affiliations:** 1 SPHeRE PhD Programme; Graduate School of Healthcare Management, RCSI University of Medicine and Health Sciences, Dublin, Leinster, Ireland; 2 Sociology; Centre for European and Eurasian Studies; Social Sciences Institute, Maynooth University, Maynooth, County Kildare, Ireland; 3 Graduate School of Healthcare Management, RCSI University of Medicine and Health Sciences, Dublin, Leinster, Ireland

**Keywords:** Solidarity, solidarities, informal solidarity, formal solidarity, collective representation, trade unions, industrial relations, camaraderie, peer-support, experiences of work, shared experiences, doctors, nurses, qualitative evidence synthesis, QES, protocol

## Abstract

Negative healthcare professional experiences of work can undermine health system stability by contributing to shortages, burnout and poor retention. Research into doctors’ and nurses’ working conditions indicates the important role of 'solidarity' to their working experience. There is limited understanding of formal (trade unions, industrial action) and informal (peer-support, camaraderie) solidarity in shaping doctors’ and nurses’ working experience. This study aims to conceptualise both forms of solidarity as perceived by doctors and nurses to understand its potential on their working experience.

This qualitative evidence synthesis (QES) protocol is reported following PRISMA-P recommendations. A search strategy has been developed using controlled vocabulary and free terms (MEDLINE, CINAHL, Scopus Embase, PubMed databases), following the Peer Review of Electronic Search Strategies (PRESS) process. English language articles will be included if they report primary, conceptually rich and contextually thick qualitative data, exploring the perceptions of informal and formal solidarity as experienced by qualified doctors and/or nurses. Its influence on the working experience, as reported by included studies, will be explored. Initial title and abstract screening, followed by full-text screening of included articles, will be completed independently by two reviewers. A grey literature search will be employed, including a targeted, domain-specific Google search of doctor and nurse national unions within ten countries with highest union density, and websites of intergovernmental organisations/agencies. Piloted data extraction forms will be used to extract study characteristics and qualitative data. The CASP (Critical Appraisals Skills Programme) tool will be used to assess the quality of included studies by two reviewers, independently. Confidence in cumulative findings will be assessed using GRADE-CERQual guidelines. The QES will be reported using eMERGe guidelines and will follow the Noblit and Hare meta-ethnography approach.

**Registration Number:** This protocol has been registered via the PROSPERO database, the International Prospective Register of Systematic Reviews. The protocol can be found on the register under the following number: CRD420251150676. Available from: https://www.crd.york.ac.uk/PROSPERO/view/CRD420251150676.

*PROSPERO Registration:* CRD420251150676

## 2.Introduction

### Introduction & background

Healthcare workforce retention is a global population health challenge. As argued by the World Health Organization (WHO), there is ‘no health without a workforce’, highlighting the need for research exploring frontline healthcare staff workplace experiences ^1^. Negative healthcare professional (HCP) experiences of work can undermine health system stability by influencing healthcare access, patient care quality and continuity – perpetuating the workforce crisis ^2^. This crisis (comprising of a labour and mental health crisis) exists internationally, and is reflected in staff shortages, burnout and poor retention ^3^. Increased workplace pressure faced by HCPs from the demands of ageing populations, and the coronavirus (COVID-19) pandemic especially, have highlighted the importance of ‘solidarity’ in shaping their working experience ^4, 5^.

Recently, the COVID-19 pandemic has highlighted two key points of relevance to health research: the exacerbation of the unfavourable working conditions (extensive working hours and unreasonable shift patterns, resource shortages, pressure, stress, unsafe staffing) faced by HCPs, as well as the role of solidarity in the management of these conditions ^4– 6^. Solidarities in work have been broadly described as a collective sense of representation, shared vision and purpose that develop within work environments. Beck and Brook ^7^ define solidarity, broadly, as a phenomenon of ‘fellow-feeling’, which develops informally in the workplace as a collective sense of togetherness and mutually shared experiences and understanding. Solidarity (especially informal) refers to peer support or camaraderie – organic and mutually beneficial interpersonal support between colleagues with a shared sense of identity ^5, 7– 10^, an example being illustrated by Korczynski’s communities of coping ^11^, where two or more healthcare practitioners support one another in the workplace.

Solidarities can also be formal – traditionally, as labour mobilisation or trade unions, commonly involving union membership of organised workers with similar material interests, ideas, or a sense of (in)justice ^7^. With respect to formal solidarity, appropriate management of labour relations via engagement of healthcare workers’ trade unions and professional representative bodies has a documented influence on addressing and safeguarding workers’ wellbeing ^3, 12^. Trade unions, e.g., the Irish Nurses and Midwives Union (INMO) and the Irish Medical Organisation (IMO) representing Irish nurses and doctors, respectively, act as key stakeholders of formal, collective representation. Having the power to negotiate reform recommendations for more sustainable working contracts, conditions and pay through industrial action, workers’ unions shape the industrial and labour relations landscape, and thus, the experience of work for doctors and nurses ^12, 13^.

In the Irish context, Byrne *et al*. ^14^ and Creese *et al*. ^15, 16^ highlight how doctors’ experiences of work are shaped by informal peer support in a situation of a perceived ‘lack of voice’. However, there is limited research available regarding informal solidarity in frontline healthcare work (with two studies specifically related to the pandemic only, taking a psychological perspective) ^5, 8^, and limited evidence coherency of how informal solidarity shapes doctors’ and nurses’ experience of their work. As with informal solidarity, there is a lack of coherency as to the perceived influence trade unions and other collective forms of representation have on the experience of work from the viewpoint of doctors and nurses ^3, 12^.

Following the consultation of the ‘RETREAT’ criteria developed by Booth *et al*. ^17^, we identified qualitative evidence synthesis as the best methodology to explore this topic and address this evidence gap. Doctors and, in particular, nurses, were chosen due to historically high unionisation rates ^18, 19^ and a tendency for these HCP unions to be single-profession(al) unions. The COVID-19 pandemic has highlighted the importance of solidarity for both professions ^4, 5^.

### Aim

This QES aims to conceptualise how doctors and nurses experience both formal and informal solidarity in work. Findings of this review can inform strategies for improving health system sustainability by addressing the workforce crisis, leveraging the priorities of the World Health Organization’s (WHO) ‘Framework for action on the health and care workforce in the WHO European Region 2023–2030’, which is to safeguard the wellbeing of staff ^1^. Additionally, a better understanding of working experiences of frontline health workers has the potential to influence workforce policy and improve retention.

### Research questions

1. What are doctors’ and nurses’ experience of formal and informal solidarity

2. How do these solidarities influence doctors’ and nurses’ experience of work.

## 3. Methods

The SPIDER ^20^ (Sample, Phenomenon of Interest, Design, Evaluation, Research Type) framework informed the review question, search terms and inclusion and exclusion criteria, which are outlined in Table 1 below. See also the ‘Study *Design*’ section, ‘ *Step 1: Getting Started*’.

**Table 1.** Research question as defined and formulated using the SPIDER framework ^20^. Each aspect of the question is mapped on to and explained in the context of each element of SPIDER, while providing a summary of study search strategy inclusion and exclusion criteria (see ‘Study Design’ below).

**S**ample	**Doctors and nurses** of **any grade/register** (including intern but **excluding undergraduate student or not fully ** **qualified groups**) or specialty, working in any healthcare setting (hospital, community, public or private etc.). Doctors and nurses can be either **active union members or non-members and** be **actively practicing or retired.** Doctors and nurses who have exited the system but have healthcare work experience will also be included. **Patient peer-support groups, or support groups for various morbidities/diseases** (either communicable or non-communicable) **will be excluded.** **Articles exploring the experiences of other allied HCPs only will be excluded**. Articles where data specifically related to the perceptions and experiences of nurses and doctors can be extracted will be included.
**P**henomenon of **I**nterest	Solidarity comprising: a. **Formal collective representation** (e.g., trade unions, unionisation & union membership - representative bodies) of doctors and nurses, most frequently involving a contractual, paid subscription to access services such as legal support/advice, organised collective bargaining, safeguarding of interests and well-being, adequate renumeration etc., via negotiation with an employer ^22, 23^. b. **Informal solidarity** (e.g., peer support, shared experiences, camaraderie), defined as voluntary, mutual(ly beneficial), organic, interpersonal support and camaraderie between peers/colleagues who have a shared sense of experience, justice, purpose, and identity ^5, 7– 10^. This can involve peer-to-peer support through institutionally organised programmes. To meet inclusion criteria, studies must specifically address either **a** or **b** above in depth, i.e., literature must include *contextually thick* and *conceptually rich* qualitative data, assessed using a QES assessment tool developed for this purpose ^24^.
**D**esign	Qualitative evidence synthesis of primary, qualitative data studies using: **- focus groups** **- interviews** **- fieldwork observations and notes** **- diaries and other qualitative data** Included literature types: - **articles** published in **peer-reviewed** journals - ‘grey’ literature (reports and websites of international, representative agencies/bodies) **Ethnographic studies**, including digital **book chapters** with thick/rich qualitative data **Excluded** research designs and literature types: - **Quantitative** primary studies - **Reviews** (qualitative or quantitative, of any nature, including systematic reviews), - **Mixed-methods** studies (meta-ethnography being applicable to qualitative studies exclusively ) - **Grey literature** in the form of **expert opinions, editorials, news articles, commentaries, abstracts in ** **proceedings, theses, dissertations.** - **Ethnographic books (hard copies** only, due to possible access restrictions and specificity to research question) - Full-text studies not written in the **English language**
**E**valuation	Experiences and perceptions
**R**esearch Type	Original qualitative research articles using designs noted above. Full-text, English language only (for the sake of preserving context/accurate interpretation of qualitative data).

### Study design

This QES will employ Noblit and Hare’s ^21^ 7-step meta-ethnography, a systematic approach to the synthesis of findings of relevant qualitative studies, translating them into one another. This provides novel interpretations, adding to the body of knowledge surrounding the phenomenon of interest; in this case, solidarity in healthcare work. The 7 steps (or ‘'phases’', as described by Noblit and Hare) include:

1. *Getting started –* identifying a novel research question or ‘intellectual interest’ which could be answered using qualitative methods (see ‘3. METHODS’ section and Table 1).
2. *Deciding what is relevant –* identifying articles, literature, and data which meet researcher-established criteria to answer the research question (described in ‘3. METHODS’).
3. *Reading the studies –* taking time to analyse and interpret the data selected in *Step 2* above (see ‘3. METHODS - Search Strategy’)
4. *Determining relatability across selected studies –* finding similarities, but also differences, across concepts, themes and author interpretations which complement or refute each other throughout the selected studies (‘3. METHODS – Data Extraction Process and Analysis & Data Synthesis’).
5. *Translating studies into one another –* analysing the data of each selected study, through line-by-line coding (creating ‘first-line constructs’), followed by the grouping of this coding into descriptive codes, based on the similarities and differences in themes and concepts present in the data (‘ *Data Synthesis*’).
6. *Translation synthesis* – generating analytical themes based on descriptive themes, as understood and interpreted by the researcher, with the help of existing theoretical frameworks to further the body of evidence related to the research question. This is also known as reciprocal translation and generates ‘second-order constructs’. Translation can also be refutational, should some constructs or concepts be conflicting (‘ *Data Synthesis*’).
7. *Synthesis expression* – achieved by discussing and reporting the synthesised, translated interpretations from Steps 4 to 6 (‘third-order constructs’) as a systematic review of included qualitative literature, while following appropriate reporting guidelines such as eMERGe ^21, 25^ (‘3. METHODS – Dissemination' and ‘4. CONCLUSION’).

The protocol for this QES is reported using the PRISMA-P protocol checklist and guidelines ^26^. The final synthesis and analysis will be reported in line with the ‘Improving reporting of meta-ethnography (eMERGe)’ guidelines ^25^.

### Eligibility criteria

Inclusion/exclusion criteria of study types, properties and study participants/population sampled are set out in the SPIDER framework ^20^ of Table 1 and addresses *‘Step 2: Deciding what is relevant’* of the 7-step Noblit and Hare meta-ethnography approach ^21^.

An English language filter will not be applied, due to the risk of excluding articles with non-English titles which include an English language translation. However, studies for which there is no available full-text English language translation will be manually removed during title/abstract screening - the rationale being the importance of context in qualitative studies, which may be impacted by translation. There will be no publication year restrictions applied to the search, the justification being that solidarity is a traditional concept in the social sciences and the likelihood of studies from the 70s and 80s in industrial relations being potentially relevant to our conceptualisation and research question.

### Search strategy

The search strategy was devised, using the PRESS Peer-Review process, to address ‘ *Step 2: Deciding what is relevant*’ of the Noblit and Hare 7-step meta-ethnography approach ^21^.

An independent, expert health librarian affiliated with the 1 ^st^ Author’s host institution was consulted to develop the search strategy and search strings for each relevant database. An initial search was developed with the help of the host institution librarian ('searcher'). A second, independent, university librarian ('reviewer') affiliated with the 2 ^nd^ Author’s host institution produced a PRESS Peer-Review ^27^ of the initial search, to finalise the search string, strategy, and increase rigour and transparency throughout the QES.

Upon meeting and discussing with both librarians, any amendments advised as part of the PRESS peer-review were clarified and implemented across the search strategy and search strings for all databases used. The finalised search strings and strategy used for the purpose of this review, as well as the PRESS peer-review form completed by both librarians, is available under the ‘Extended Data’ section. The PRESS peer-review proved to be a very beneficial exercise, both in formalising the search strategy, as well as increasing the rigour and transparency of study search methodology. Additionally, both librarians offered invaluable amendments and suggestions for a more precise database search by pointing to some minor omissions and errors.

Preliminary search terms were developed to reflect the research question, using both keywords and controlled vocabulary of MeSH terms. MeSH terms were used for term refinement. The final search string will be translated and applied to MEDLINE (Ovid; doctor focus), CINAHL (Cochrane; nurse focus), Scopus (Elsevier; a multidisciplinary database, with coverage of social sciences and humanities research), and Embase (Ovid/Elsevier). PROSPERO will be searched for similar, prospective reviews. Article authors may be contacted for clarifications.

Search results will be subject to initial title and abstract screening carried out independently by two reviewers and selected based on relevance and meeting inclusion criteria outlined above. Disagreements and conflicts will be resolved by consulting a third reviewer.

***Grey literature search strategy.*** A two-step, grey literature search will be carried out via a targeted, domain specific Google search using the following keywords: “nurse”, “doctor”, “physician”, “midwife”, “frontline worker”, “union”, “experience”, “solidarity”, “peer support”, “employee morale”, “workplace support”, “strike”, “collective organisation”, “camaraderie” of:

a) doctor and nurse-specific unions in the ten countries with the highest union density as per OECD data ^28^: Iceland, Sweden, Denmark, Finland, Norway, Belgium, Italy, Luxembourg, Canada, Ireland. It is hoped that countries where union density and activity is highest will have published reports on union-member experiences.

b) the websites of the following intergovernmental organisations and agencies will also be searched: Eurofound, WHO, OECD (Organisation for Economic Co-operation and Development), ILO (International Labour Organization) for key data relevant to the research question and inclusion criteria.

The reference list of all included documents will be screened for additional studies using forward and backward citation (citation chasing via ‘Citationchaser’), supplemented by hand-searching reference lists where necessary ^29^.

***Screening/data management & selection process.*** Following the search, all identified citations will be collated and imported into Zotero. After deduplication, citations will be uploaded into Covidence. The title and abstracts will be screened independently by two reviewers against the inclusion criteria outlined in Table 1.

The senior author will review the first 100 titles/abstracts as part of a pilot test. Disagreements will be resolved by consulting a third reviewer.

Remaining articles will be subject to independent, full-text screening by two reviewers - disagreements will be resolved by consulting a third reviewer ( *Steps 2 and 3* of the meta-ethnography approach) ^21^.

### Data extraction process and analysis

For the purpose of collecting both descriptive characteristics of studies included in this review, as well as qualitative data reported in each, data extraction forms will be drafted, piloted, and finalised. Data will be extracted using said preconceived extraction forms and the qualitative data (such as interview or focus group quotes/statements, observational notes and author analysis) generated from each included study will be imported into the latest NVivo software available to the 1 ^st^ Author, for further synthesis and analysis. This will be done independently by MD, with review from JPB – disagreements, should they arise, will be resolved by consulting SOR. This will address *Steps 3 and 4* of the 7-step meta-ethnography process ^21^.

***Data items.*** Data items collected will reflect the requirements proposed in data extraction forms. This will include:

- Sample demographics and sample size.

- Country of origin of each study.

- Year of publication.

- Author name(s).

- Qualitative data generated and the authors’ interpretation of this data. This could include sections/paragraphs or transcripts, or direct study participant quotes interpretations, and analyses from grey literature.

### Data synthesis

Meta-ethnography as described in the 7-step process developed by Noblit and Hare will be used to analyse the data, through line-by-line (first-order constructs) coding ( *Step 4* of the process), followed by first-order grouping into descriptive codes (second-order constructs, including any contradictory findings, addressing *Step 5* of the process), to generate analytical themes (third-order, using any existing conceptual or theoretical frameworks in this specific research field) ^21^. In essence, this is a method of interpreting interpretations of interpretations to synthesise common or conflicting findings through analytical themes. Analytical themes will be translated into the results of individual studies, which adds cohesiveness to pre-existing knowledge, and can summarise how knowledge from one existing study can relate to another ( *Step 6*) ^21^.

***Outcomes & prioritisation.*** In line with the research question and qualitative nature of this review, primary data will include doctors’ and nurses’ experiences and perceptions of formal and informal solidarity.

Secondary data (which will be inductive) will explore the impact of this solidarity experience, e.g., staff expressions of engagement, intention to leave current post or system, workplace satisfaction, doctors’/nurses’ satisfaction with the representativeness of trade unions/consequences of industrial action, and the perceived ability of informal and formal solidarity to enforce/promote/enable workplace wellbeing and voice concerns.

### Risk of bias in individual studies, meta bias(es)

Studies will be independently assessed for risk of bias (in both study methodology and outcomes reporting) by MD and JPB (with input from SOR should discrepancies arise) using the ’Critical Appraisal Skills Programme (CASP) Qualitative Studies Checklist’ ^30^. Studies will not be excluded or included based on quality, but as previously stated, based on the richness and thickness of qualitative data. Contextual thickness and conceptual richness will be determined using a QES assessment tool developed by Ames *et al*. ^24^.

### Confidence in cumulative evidence

To assess the overall level of confidence to be placed in the overall findings of this review, and to increase reporting transparency, the (GRADE-CERQual) approach ^31^ will be applied to the cumulative evidence synthesis. Confidence in the cumulative evidence will be graded, assessed independently by each author and will be discussed by the research team.

### Reflexivity

To ensure an unbiased approach, especially during the data analysis and the presentation of study findings, the research team will reflect on individual preconceptions and past experiences which could interfere with the validity of this study. The 1 ^st^ Author (MD) has a background in microbiology and public health, with some experience in the administrative aspect of healthcare management and teaching, but little experience in sociology or industrial relations. Two of the remaining authors (JPB and SOR) have an extensive background in sociology, with experience in the field of healthcare management. JPB’s previous work has largely focused on the working conditions and experiences of frontline healthcare workers (especially doctors). All authors have carried out research in both an Irish and an international context. However, none of the authors are qualified doctors, nurses, or any other allied healthcare professionals. The research team will reflect on any possible preconceptions at each stage of producing this review, which may affect the interpretation of data and research results.

### Dissemination of information

It is hoped that this review will be published in a relevant peer-reviewed journal. The published review and its findings will be incorporated as part of the 1 ^st^ Author’s PhD research and disseminated at relevant conferences, PhD research showcases (internal and external to the 1 ^st^ Author’s host institution) and presented to policymakers and stakeholders of interest as relevant (‘ *Step 7: Synthesis Expression*’) ^21^.

## 4. Discussion

COVID-19 highlighted a number of key points: there is no healthcare without a health workforce ^1^, already strained working conditions and staff wellbeing were exacerbated by the pandemic experience, and solidarity was a key influence on the experience of healthcare delivery ^5, 6, 8– 10^. The findings of this QES can inform strategies for improving health workforce sustainability by leveraging the priorities of the WHO’s 'Framework for action on the health and care workforce in the WHO European Region 2023–2030’ to safeguard the wellbeing of health workforce staff ^3^. A better understanding of the working experiences of frontline health workers, and the role of solidarity on this experience, has the potential to inform health workforce retention strategies and policies.

## 5. Limitations, final considerations

### Limitations

This review will examine the experiences of qualified doctors and nurses only – undergraduate student doctors and nurses, and other allied healthcare professionals (be they students or fully qualified professionals) will not be considered. Therefore, this review will not capture the phenomena of solidarity, formal or informal, as perceived by other types of HCPs, or future frontline health workers. Due to the current political, social, and industrial relations landscape, views of those who practice in countries where trade union membership is considered unprofessional, taboo, or perceived to have consequences for one’s employment security (e.g. USA currently – especially in terms of the possibility of newly emerging research being limited, Belarus, Hungary, Turkiye) may not be captured fully despite this study aiming to capture an international perspective. Research emerging from countries where union density tends to be high (for example, Nordic and Benelux countries), may be overrepresented in this review.

Additionally, articles not published in the English language are excluded, which, despite all efforts to include international literature, does not fully encapsulate the international perspective of doctors and nurses surrounding the concept of solidarity in the workplace. Although meta-ethnography is a powerful evidence synthesis tool for qualitative research, it often necessitates an exclusion of mixed-methods and quantitative studies such as research which utilises questionnaires or surveys with an explanatory or open-text element. Therefore, “thinner” qualitative data and quantitative data fall outside the scope of this review. However, this is partially justified with the primary aim of meta-ethnography being the synthesis of valuable rich and thick qualitative data only, which tends to be reported on and generated more transparently and rigorously in a fully qualitative study, as opposed to being an adjunct of a primarily quantitative study (which oftentimes yields ‘thin’ qualitative data).

## Study status

On publication of this protocol, the review has commenced with the initial title and abstract screening phase.

## Data Availability

No data associated with this article. PORSPERO Registration: **https://www.crd.york.ac.uk/PROSPERO/view/CRD420251150676**. The following extended data is available for this protocol under the Open Science Framework and the terms of the Creative Commons Zero “No rights reserved” data waiver (CC0 1.0 Public domain dedication): https://doi.org/10.17605/OSF.IO/DGMK4 ^32^. Preliminary Search Strategy.docx (Provisional search string, exploring relevant MeSH terms prior to peer-review by a second, independent librarian.) PRESS Peer-Review Form.docx (Fully completed search string and search strategy peer-review document completed by librarian one and two.) Finalised Search Strategy.docx (Full, peer-reviewed search string applied as part of the search strategy, applied to databases PubMed, MEDLINE, CINAHL, Scopus and Embase) PRISMA-P 2015 Checklist ^26^ for ‘Conceptualising doctors’ and nurses’ experience of formal and informal solidarity: A Meta-Ethnography Protocol’ is available as extended data as ‘PRISMA-P Checklist’.docx under the Open Science Framework and the terms of the Creative Commons Zero “No rights reserved” data waiver (CC0 1.0 Public domain dedication): https://doi.org/10.17605/OSF.IO/DGMK4 ^32^.
