# Supplementary material for: Conceptualising doctors’ and nurses’ experience of formal and informal solidarity: A Meta-Ethnography Protocol
Source: HRB Open Res. 2026 Mar 10;8:128. Originally published 2025 Dec 5. [Version 2] doi: 10.12688/hrbopenres.14294.2 (PMC13231968; doi:10.12688/hrbopenres.14294.2)
Supplement: Peer review history

# Reviewer report: Reviewer response for version 2

**DOI:** 10.21956/hrbopenres.15818.r54067

**Published:** 2026-03-19

**Authors:** Brenna Doran

**Affiliations:** 1 University of California San Francisco, San Francisco, Canada

The authors have systematically addressed the concerns raised in the initial review. The clarification of the meta-ethnography 'audit trail' and the integration of specific sociological frameworks (Social Exchange Theory, Durkheim) significantly strengthens the protocol's theoretical foundation. While the exclusion of Allied Health Professionals remains a limitation regarding the understanding of multi-disciplinary informal solidarity, the authors have adequately justified this via project feasibility and have appropriately noted this in the limitations section. I am satisfied with the methodological refinements.

Is the study design appropriate for the research question?

Partly

Is the rationale for, and objectives of, the study clearly described?

Partly

Are sufficient details of the methods provided to allow replication by others?

Partly

Are the datasets clearly presented in a useable and accessible format?

Not applicable

Reviewer Expertise:

My research and professional expertise focus on the operational challenges and psychological well-being of the infection prevention workforce, with specific emphasis on the following topics: Infection preventionist staffing and workload, Healthcare worker burnout and attrition, Professional resilience and leadership coaching, Retention factors in specialized healthcare roles, Infectious disease epidemiology and clinical microbiology, and Change management and patient safety advocacy.

I confirm that I have read this submission and believe that I have an appropriate level of expertise to confirm that it is of an acceptable scientific standard.

# Reviewer report: Reviewer response for version 1

**DOI:** 10.21956/hrbopenres.15731.r52942

**Published:** 2026-01-29

**Authors:** Brenna Doran

**Affiliations:** 1 University of California San Francisco, San Francisco, Canada

**Article Summary**

 This protocol outlines a Qualitative Evidence Synthesis (QES) designed to conceptualize formal and informal "solidarity" among qualified doctors and nurses. The study focuses on how these solidarities influence the working experience and potentially address health system instability and workforce retention issues. The methodology follows the Noblit and Hare seven-step meta-ethnography approach, utilizing a search strategy refined by the PRESS process across major databases (MEDLINE, CINAHL, Scopus, Embase, and PubMed). The authors plan to utilize the SPIDER framework for criteria definition, CASP for quality appraisal, and GRADE-CERQual for assessing confidence in the cumulative findings.

**Overall Strengths:**

- The manuscript addresses a timely and significant topic—healthcare workforce retention and the role of solidarity.
- The use of the PRESS process and professional librarians makes the search strategy highly transparent and reproducible.
- Using Noblit and Hare’s meta-ethnography and the SPIDER framework provides a solid academic structure for the study.

**Is the rationale for, and objectives of, the study clearly described? Answer: Partly**

 While the manuscript identifies a significant gap regarding healthcare workforce retention, the rationale would benefit from further strengthening.

1. For primary research terms such as “negative experiences” and “solidarity,” recommend using the definitions from the studies being referenced unless the intent is to provide a novel definition. If a novel definition is intended, this must be clearly communicated.
2. Recommend ensuring the use of international references aligns with the stated local focus and application of the findings.
3. Recommend stating if the goal of "conceptualizing" solidarity is to create a brand-new theory or to refine an existing one.
4. Recommend including terms like "Medical Sociology" or "Industrial Relations" to improve the study's discoverability.
5. The phrase "negative experiences" is broad. Recommend adding context regarding the root causes of these experiences and the role of the work environment.
6. Recommend defining the "workplace crisis" more clearly (e.g., chronic staff shortages, increased patient mortality) and supporting it with appropriate citations.
7. Ref 2 primarily identifies burnout as the issue. Recommend clarifying if burnout is the primary focus and ensuring the terminology used in the paper aligns with the cited references.
8. Ref 2 is from 2014 and is based on a US study. Depending on the intended scope, recommend adding more current studies and references that reflect the appropriate geographical and historical scope to demonstrate the chronic nature of the issue.
9. The paper states the crisis exists internationally; however, Reference 3 appears to be an opinion article focused on Europe that cites other studies. Recommend citing the original research studies and including a broader international lens to support the "labour and mental health crisis" claim.
10. As "solidarity" is a primary variable, recommend clearly defining it and explaining how it relates to the specific problem being addressed. The current text provides multiple definitions; recommend specifying which one the reader is to follow.
11. There is a heavy reliance on references from Ireland. If the Irish context is unique or superior in its research on solidarity, recommend providing that context.
12. There is a disconnect between the global scope (WHO) and the specific geographical scope (10 OECD countries). If the research is limited to these 10 countries, recommend reconsidering the references in the introduction to support that specific scope.
13. The specific problem and research question require greater clarity. Recommend explaining *why* a lack of knowledge regarding solidarity is a problem. For example: The workforce crisis harms retention → Solidarity may be the "glue" that prevents quitting → We do not know if staff perceive unions or peer support as effective → Therefore, this study is needed to inform policy.
14. Recommend further elaborating on the tensions between informal solidarity, formal solidarity, and the role of the union, as these remain unclear.
15. Regarding the use of the RETREAT methodology, it is unclear if there are enough primary studies to synthesize. Recommend addressing this concern, which is reinforced by the absence of robust references in the section outlining the research problem.
16. To strengthen the introduction, recommend considering the following:

- Define the “so what” of the problem (e.g., “we don't know if solidarity is enough to stop doctors and nurses from leaving”).
- Explicitly state the objective around the third paragraph using a phrase like: "The objective of this Qualitative Evidence Synthesis is to..."
- State the research question clearly (e.g., "How do doctors and nurses perceive the influence of both informal peer support and formal union representation on their decision to remain in the workforce?").
- Streamline the definitions of solidarity to focus on why the study is necessary.

**Is the study design appropriate for the research question? Answer: Partly**

1. This section outlines the steps of the meta-ethnography method without clearly explaining how it will be applied to this specific study. Recommend adding a justification for why these specific steps are the most appropriate tools to address the "solidarity" problem.
2. The author describes the seven steps of Noblit and Hare in a general sense, but the section lacks a clear "audit trail."  Specifically, how will the author ensure that ‘third-order' interpretations remain grounded in the original contexts of the international studies. Example (1 ^st^ order: The raw quotes from the doctors/nurses in the original studies, 2nd order: The original researchers' interpretations of those quotes, and 3 ^rd^ order the new synthesis of those interpretations.) This will also help the reader understand how the author will address a potential limited size of original research papers.
3. Providing this detail will also help the reader understand how the author will address the potential issue of a limited number of original research papers.
4. There is a heavy use of circular referencing (e.g., multiple references to Section 3 and Table 1). While referencing is appropriate, recommend streamlining these mentions to avoid repetitive citations.
5. The author states that an English filter will not be applied to avoid excluding translated articles, yet also states that studies without English full-text will be manually removed. Recommend clarifying this contradiction; excluding non-English studies may result in missing key international research.

**Are sufficient details of the methods provided to allow replication by others? Answer: Partly**

1. The historical scope (dating back to 1970) appears broader than the background and research problem, which focus on COVID-19 and modern aging populations. If historical studies are to be included, recommend adding an explanation of how contexts from the 1970s forward remain relevant to the current healthcare environment and modern union relationships.
2. Regarding the sample, recommend clarifying the specific criteria for "doctor" and "nurse" from an international perspective (e.g., education and licensure). For example, in the US, "doctor" can refer to many terminal degrees (MD, DNP, PhD); recommend specifying if the study is limited to physicians (MD/DO) and how allied health professionals with similar degrees will be handled.
3. Recommend clarifying the definition of "experiences" in the context of the exclusion criteria for allied healthcare providers. Excluding frontline staff such as respiratory therapists, surgical technicians, and pharmacists may result in missing impactful studies, as "solidarity" is often a multi-disciplinary phenomenon.
4. The exclusion of "patient peer-support groups" is noted; recommend providing a clear justification for why this specific group is being excluded from a study on workplace solidarity.
5. High-quality healthcare studies often use mixed-method approaches to show both what is happening (quantitative) and why (qualitative). By excluding these entirely, the author may miss "thick and rich" qualitative data. Recommend the author justify why the qualitative components of mixed-methods studies are considered insufficient for this specific synthesis.
6. Most current keywords focus on "strikes" and "unions." Recommend adding sociological terms like "professional identity," "occupational community," or "social capital" to find studies on informal support that the current industrial relations terms might miss.
7. Since this is a social topic, consider searching databases like PsycINFO or SocINDEX.
8. As Table One includes excluding non-english full-texts please clarify if the author will be searching for non-english terms. If search is limited to English Keywords, the ‘translation’ of the string (for grey searches) is purely technical rather than linguistic and needs to be specified.
9. When two people test the first 100 articles, recommend using a math-based score (like Cohen’s Kappa) to prove both reviewers are truly aligned on the study criteria before proceeding.
10. In a meta-ethnography, it is vital to distinguish between 1st-order constructs (participant quotes) and 2nd-order constructs (original author interpretations) during extraction. Recommend explicitly stating how the extraction form will separate these to ensure an organized translation process.
11. The author mentions using a QES assessment tool (Ref 24) but does not explain when the quality appraisal occurs. Recommend clarifying the timing of this step (e.g., during full-text screening or after extraction) and at what point "thin" studies are discarded.
12. The author defines 1st-order constructs as "line-by-line coding" and 2nd-order as "descriptive codes." Recommend clarifying whether the coding is being applied to the raw data or the original researchers' analysis.
13. The author mentions that analytical themes will be "translated into the results of the individual studies." As Step 6 is typically the reverse—where individual studies are translated into one another to create themes—recommend confirming the order and ensuring the synthesis emerges from the studies.
14. Regarding 3rd-order constructs, recommend naming the "existing conceptual or theoretical frameworks" being considered to ensure the interpretations have a strong theoretical grounding.
15. The protocol mentions "refutational translation" but lacks an execution plan. Recommend detailing how the team will handle "disconfirming cases" (e.g., where formal solidarity undermines informal camaraderie) to avoid a homogenized result that ignores contextual tensions.
16. It is unclear how the author will distinguish between the "experience" (primary data) and "impact" (secondary data) of solidarity, as these are often linked in narratives. Recommend justifying the prioritization of these data items, particularly given that "intention to leave" is a core problem identified in the introduction.
17. Regarding the GRADE-CERQual approach, recommend clarifying how the four components (Methodological Limitations, Relevance, Coherence, and Adequacy) will be operationalized. Specifically, recommend defining "Adequacy"—whether it is based on the number of studies or the "thickness" of the data.
18. Recommend specifying if the CERQual assessment will be performed independently by two reviewers to reduce subjective bias and increase confidence in the cumulative findings.
19. The discussion focuses heavily on the COVID-19 pandemic and the WHO Framework. Recommend explicitly connecting the potential findings of this QES to specific policy levers beyond "wellbeing," such as how solidarity might practically influence retention strategies.
20. The author acknowledges that countries where unionization is "taboo" may be underrepresented. Recommend clarifying if the search strategy will proactively look for "alternative" forms of solidarity in these regions to ensure the study is truly international as claimed.
21. The limitation regarding the exclusion of "allied healthcare professionals" is noted. However, since the author justifies this by focusing on high unionization rates in nursing and medicine, recommend discussing how the exclusion of the "wider team" might limit the understanding of "informal solidarity" (camaraderie), which is often multi-disciplinary.
22. The author justifies the exclusion of allied healthcare professionals by citing the high unionization rates of doctors and nurses. However, since the study aims to explore both unionized and non-unionized staff, this justification appears inconsistent. Furthermore, because "informal solidarity" (camaraderie) is inherently multi-disciplinary, excluding the "wider team" may result in a fragmented or incomplete understanding of how doctors and nurses experience workplace support. Recommend the author clarify this exclusion, especially since unionization structures and professional roles vary significantly across the ten countries included in the study.

Is the study design appropriate for the research question?

Partly

Is the rationale for, and objectives of, the study clearly described?

Partly

Are sufficient details of the methods provided to allow replication by others?

Partly

Are the datasets clearly presented in a useable and accessible format?

Not applicable

Reviewer Expertise:

My research and professional expertise focus on the operational challenges and psychological well-being of the infection prevention workforce, with specific emphasis on the following topics: Infection preventionist staffing and workload, Healthcare worker burnout and attrition, Professional resilience and leadership coaching, Retention factors in specialized healthcare roles, Infectious disease epidemiology and clinical microbiology, and Change management and patient safety advocacy.

I confirm that I have read this submission and believe that I have an appropriate level of expertise to confirm that it is of an acceptable scientific standard, however I have significant reservations, as outlined above.

# Response

**Published:** 2026-02-14

**Authors:** Maja Dumana

**Affiliations:** SPHeRE PhD Programme, GSM, Royal College of Surgeons in Ireland, Dublin, Leinster, Ireland

Thank you kindly for taking the time to publish your peer-review, it is much appreciated. 

***Is the rationale for, and objectives of, the study clearly described? ***

1. Thank you for this comment. We have now clarified what is meant by ‘’negative experiences’’ in the first paragraph of the ‘Introduction and background’ section. Additionally, we define solidarity – based on the references listed and discussed, in Table 1 (SPIDER). This definition also drives our search strategy. We have also clarified that the purpose of this study, in line with a meta-ethnography approach, is to produce a novel conceptualisation of the role of solidarity for doctors and nurses and their working experience. 
2. Thanks for this suggestion. In the final QES paper, we will ensure a balance between both national and international references. We refer to the Irish context in the background especially but make it clear that our search strategy and overall study scope is international literature. We have also described the ‘’healthcare workforce crisis’’ as an international phenomenon, with global evidence (WHO). We have also included evidence from countries other than Ireland, to expand the context.
3. Thank you. As per the meta-ethnography approach, our primary goal is to develop a new theory of solidarity’s impact for doctors and nurses. This point has been clarified in the protocol aim. 
4. We really welcome this suggestion – we have added ‘’Medical Sociology’’ as a term to the protocol keywords. The term ‘’Industrial Relations’’ has already been included. 
5. Thank you, we have now clarified what is meant by ‘’negative experiences’’ (please refer to point 1 above).  
6. Thank you for this comment. References 3 and 4 provide core features defining the ‘’workforce crisis’’. These features have now been clarified in the ‘Introduction and background’ section.  
7. Thank you – we have reviewed the protocol to ensure there is no overemphasis on burnout, as it is not the sole focus of our research. Reference 2 uses burnout as one of many outcomes or issues facing healthcare staff, but focuses more on illustrating the importance of considering working lives, experiences and conditions in health systems research.  
8. Thank you very much for this suggestion. We have edited this section of the ‘Introduction and background’ to include more recent EU and UK references. We have highlighted the range of work stressors and conditions experiences by healthcare staff. Please also refer to point 7 above.  
9. Thank you. We have now expanded the citations to include various primary studies illustrating poor working conditions and mental health issues for doctors and nurses as evidence of the international crisis. 
10. Thank you. The guiding definition in our research is provided in Table 1. However, to improve clarity, we have added ‘’Please see Table 1 for specific definitions of solidarity which will guide this study.’’  in the ‘Background and introduction’’ section and the ‘Methods’ section. 
11. Thank you for this comment. To better describe an international context for the issue at heart, as well as solidarity for both doctors and nurses, more references have now been added (please see point 8 and 9). Regarding union bodies cited in the protocol, national (Irish) examples have been used for formal solidarity, as these are the most familiar to the research team. However, these bodies represent standard trade union ethos, membership and engage in activity common to many typical trade unions globally.  
12. To clarify, the study is not limited to these 10 countries, and it is made clear that this focus is part of the grey literature search only, (the main literature search has no geographical limits as stated).The countries were chosen as they have the highest general workforce unionisation rates (according to the OECD). This data may not have been picked up by the standard search otherwise.  
13. Thank you for this suggestion. We have now edited the problem and research question in the ‘Background and introduction’’ to read as follows:  
  ‘’As established, negative work experiences have the potential to exacerbate the workforce crisis by undermining HCP retention, and contributing to burnout and shortages. Meanwhile research has shown that solidarity shapes the working experience of HCPs, particularly that of doctors and nurses, profoundly. However, there is no universal consensus on whether doctors and nurses perceive both informal solidarity or formal representation (mainly trade union support) as effective in mediating negative work experiences. It is worthwhile, therefore, to synthesise existing literature exploring these perceptions to better inform and support health workforce policies.’’ 
14. Thank you for this suggestion. We have now mentioned defining the relationship/tensions between formal and informal solidarity in the ‘Outcomes’ section.   
  However, these tensions do remain unclear in the literature. To our knowledge, no review provides any context as to this relationship for doctors and nurses. This relationship is something that our QES has potential to contribute to. Additionally, this will be examined in a further study as part of my PhD project (qualitative, semi-structured interviews with union and non-union member doctors and nurses).  
15. Thank you. Additional studies have now been cited which highlight the problem. QES meta-ethnography is not concerned with a large number of studies, but rather, the richness and thickness the data of the included studies contributes to the overall synthesis. In fact, a large number of studies may interfere with accurate synthesis,and may warrant employing a sampling of studies to mitigate this ( Sattar et al., 2021)(Reference 37 in the updated manuscript). Sources of data are being assessed using the QES meta-ethnography data richness and thickness tool (Ref 29 in the updated manuscript).
16. Thank you. We have now stated that it is not clear how exactly solidarity shapes the working experience of doctors or nurses, and thus, warrants review. This has been done towards the end of the ‘Background and introduction’ section.
  The objective of the QES has been re-emphasised towards the very end of the ‘Background and introduction’ section.  
  Our research question is stated in the ‘Aims - Research Question’ section. To model your suggestion, we have edited this section to read as only one coherent question.
  The definitions of solidarity have been defined in Table 1.  

***Is the study design appropriate for the research question?***

1. The use of QES has been justified throughout the protocol. Each of the 7-steps of meta-ethnography are contextualised with respects to the study processes. Each relevant research step described as part of this study is referenced back to one of the seven meta-ethnography steps (e.g. ‘’Step 3 - ‘’Reading the studies’’ ‘’...’’ (see ‘3 - METHODS - ‘Search Strategy’)’’. In line with your suggestion, we have also strengthened our justification for addressing the research question with the use of meta-ethnography, as developed by Noblit and Hare, in the first paragraph of the ‘Study Design’ section (‘3.Methods’). 
2. Thanks for this comment. We have now clarified how data extraction and analysis will be managed to account for three orders of data. This has been clarified in the ‘Study Design’, ‘Data Extraction and Analysis’ and ‘Data Synthesis’ subsections of the ‘Methods’ section.  
3. Please refer back to my comments in point 15 above, as I feel like they may also be applicable to this suggestion.  
4. Thank you for this suggestion. We have amended some references to read, e.g. ‘’Please refer to the METHODS section, specifically...’’.
5. We appreciate this suggestion. We have now better clarified this. Studies for which there is no English language translation will be excluded. However, to avoid excluding studies with non-English titles which are otherwise supplemented with an original English translation (provided by the authors/journal), no English language filter will be applied to the search. Studies not written in English (title or otherwise), without a full-text English translation from the authors of the study or the publishing journal, will be excluded manually.  
  We have also already included a justification for this exclusion. Qualitative study findings, especially when discussing experiences and perceptions via e.g. direct study participant quotes, can easily lose context (linguistically, in this case), when accurate translation is not provided at the original source. 
  We have also listed excluding studies not written in the English language as a limitation. 

***Are sufficient details of the methods provided to allow replication by others?***

1. Thank you for this suggestion. We have now stated the following in the ‘Eligibility Criteria’ section. This reads as follows:   
  ‘’Although previously cited studies describe solidarity which may be more recent (e.g. in the context of the COVID-19 pandemic), the relevance and impact of solidarity for both doctors and nurses is a historically relevant concept. Therefore, in order to broadly address the research question, to note any historical differences in sentiment towards solidarity, and to minimise the risk of excluding older, but highly relevant literature, no publication year restriction filter will be applied.’’  
  As mentioned above, solidarity is a historically intricate phenomenon which evolves throughout the year across professions and culture ( Wildt, 1999).
2. We have now re-emphasised that the study is limited to medical, qualified (licensed) doctors (i.e. not doctorates, PhDs or similar) and nurses.  
  We have also clarified that, in order to capture international spelling variants and titles, MeSH terms, other database specific vocabulary and appropriate terms and truncations (e.g. ‘’physician*’’) have already been used with respects to the search strategy.  
3. Thank you for the comment. We absolutely acknowledge and agree that solidarity is often a multi-disciplinary phenomenon. However, we justify the decision to exclude other allied HCPs mainly due to the fact that these professionals tend to hold membership in multi-profession union as opposed to mostly single-profession unions, such as doctors and nurses. Also, due to this being a PhD research project QES, including other allied healthcare professionals would stretch the scope and feasibility of the study. We also discuss this in the limitations section of the protocol. 
  Future planned research as part of the PhD project will involve qualitative interviews with doctors and nurses discussing solidarity where its multi-disciplinary nature can be explained further.
4. Thank you – we have made the rationale for this exclusion in Table 1. This exclusion is primarily to limit the number of irrelevant search result being returned.  
5. The exclusion of mixed-methods studies and quantitative studies has been justified in the protocol as follows:  
  ‘’Although meta-ethnography is a powerful evidence synthesis tool for qualitative research, it often necessitates an exclusion of mixed-methods and quantitative studies such as research which utilises questionnaires or surveys with an explanatory or open-text element. Therefore, “thinner” qualitative data and quantitative data fall outside the scope of this review. However, this is partially justified with the primary aim of meta-ethnography being the synthesis of valuable rich and thick qualitative data only, which tends to be reported on and generated more transparently and rigorously in a fully qualitative study, as opposed to being an adjunct of a primarily quantitative study (which oftentimes yields ‘thin’ qualitative data (Ames et al., 2024 ; Noblit and Hare, 1988)’’ (Ref 28, 29 in the updated manuscript). 
  The ‘Limitations’ section also lists this exclusion, which the research team deems necessary in order to follow the gold standard approach to conducting meta-ethnography.
6. Thank you for this recommendation - ‘’social capital’’ in particular is a worthwhile term to matching our search criteria. However, the current search strategy once developed and piloted has captured articles exploring this topic or referencing this particular term. Terms such as ‘’community’’ have already been included in the search strategy. Terms similar to ‘’professional identity’’ and ‘’occupational community’’ have shown to generate a large number of irrelevant results when previously piloted, and are not specific enough for the purpose of addressing the research question
7. Thank you for this suggestion. In order to explore this topic from a social sciences perspective, we have already included the database Scopus, which provides context from a social sciences and humanities perspective, the database focus being largely multi-disciplinary. 
8. Thank you – we have clarified that the author will not be searching for non-English terms.  As already mentioned, truncations and terms such as ‘’physician*’’ have already been included in the search string.We have now stated that there will be no translation of search strings or search terms for neither search.  
9. Thanks for this suggestion. However, we will not use Cohen’s Kappa or other mathematical score to evaluate inter-rater reliability. As per Smith and McGannon (2017), the use of inter-rater reliability is in conflict with qualitative methods: 
  ‘’ Member checking and inter-rater reliability are shown to be ineffective for verification, trustworthiness, or reliability purposes.’’ 
   Instead, two reviewers will review the first 100 titles/abstracts as part of a pilot test. The team will discuss any conflicts. Final disagreements will be resolved by a third reviewer.
10. Thank you for this suggestion. We have now addressed this (please refer back to point 2 of the previous section of comments). 
11. Thank you. We have now clarified the stages at which the QES assessment tool developed by Ames et al. will be implemented in the ‘Screening/Data Management & Selection’ section of the protocol. We will also include a reasoning trail for all decisions made using the tool developed by Ames et al. (Ref 28 in the updated manuscript) for screening purposes as part of the full QES publication.
12. Thank you for this suggestion. To clarify, we are applying coding (line-by-line coding, and creating descriptive codes) to both the raw data from participants and the original researcher’s analysis from primary studies. We have amended the protocol to reflect this.
13. Thank you for this clarification, this has been amended. Individual study results (i.e. raw data, authors’ interpretations) will be translated into one another to create/synthesise novel themes, adding to the body of knowledge i.e. new interpretations of author’s interpretations of primary data. This has now been stated in the protocol.
14. Thank you for this suggestion. We will keep this in mind at later stages as this will be an iterative process. Some potentially relevant theoretical frameworks have been proposed, including the Social Exchange Theory and Durkheim’s Sociological Foundations (mechanical and organic solidarity) (Ref 35 and 36 in the updated manuscript).
15. Thank you for this comment. We have now addressed this (please refer back to point 10 above and point 2 of the previous section of comments).  
16. Thank you for this suggestion. We have now clarified that there is no hierarchy (i.e. no primary or secondary data). This decision came from discussing with the research team. Experience and impact are equally important, related and not easy to differentiate. Additionally, our research question sets out to address both the experience and impact/influence of solidarity. The ‘’data’’ referred to here refers to outcomes.  
  Additionally, please refer to point 2 from the previous section, and points 10, 12 and 15 from this section for a clarification on how data will be managed.  
17. Thank you for this suggestion. We have now elaborated on this approach in the ‘Confidence in Cumulative Evidence’ section. Please also refer to points 11 and 5 directly above, and point 15 of the previous section).  
18. We have amended this in the protocol to emphasise that this assessment will be carried out independently by MD and JPB, and will be discussed by the team.  
19. Thank you for this suggestion. This will be addressed more comprehensively in the full QES paper. However, we have amended the ‘Discussion’ section to add an explanation stating that representative bodies and organisation will be informed of key findings regarding how solidarity can encourage workforce retention, amplify employee voice and belonging, and address some elements of the workforce crisis.  
20. Thank you for this suggestion. The purpose of this point in the limitations section was ensure transparency and highlight we are aware of limitations. Specific regions will not be targeted for more covert forms of solidarity. As mentioned, in order to capture an international perspective, our search strategy has been designed using broad terms (e.g. ‘’doctor*’’ but also ‘’physician*’’) with appropriate truncations and database specific controlled vocabulary. 
21. Thank you for this comment. Please refer to my response in point 3 above, as there is some overlap. Focusing on specific professions (i.e. doctors and nurses) will offer our study clarity and feasibility. Additionally, qualitative interviews with doctors and nurses, which will take place as part of a different study under this PhD project, will aim to capture a multi-disciplinary experience.
22. Thank you for this comment.  
  Please refer to point 21 and point 3 above, as my responses are also relevant here. A profession specific focus will allow for the exploration of formal and informal solidarity within the same profession, which is not inconsistent in terms of what has been set out by the research question. Theorising connections between union rates of nurses and camaraderie for other allied healthcare professionals would not necessarily provide a coherent answer to the research question.  
  The scope of this study also requires feasibility – hence, the focus on doctors and nurses. As addressed in point 12 of the previous section of comments, the 10 countries mentioned are related to the grey literature search only.  

# Reviewer report: Reviewer response for version 1

**DOI:** 10.21956/hrbopenres.15731.r52933

**Published:** 2026-01-29

**Authors:** Jane Chudleigh

**Affiliations:** 1 King's College London, London, England, UK

Thank you for submitting this protocol for a review conceptualizing doctors' and nurses' experiences of formal and informal solidarity. 

 The protocol is very well written. 

 I only have one minor suggestion/query; might you be able to state in the screening section which reviewers (initials) will be reviewing and resolving conflicts (similar to the section on risk of bias)?

Is the study design appropriate for the research question?

Yes

Is the rationale for, and objectives of, the study clearly described?

Yes

Are sufficient details of the methods provided to allow replication by others?

Yes

Are the datasets clearly presented in a useable and accessible format?

Not applicable

Reviewer Expertise:

Screening, genetics, communication of genetic risk, cystic fibrosis, sickle cell.

I confirm that I have read this submission and believe that I have an appropriate level of expertise to confirm that it is of an acceptable scientific standard.

# Response

**Published:** 2026-02-14

**Authors:** Maja Dumana

**Affiliations:** SPHeRE PhD Programme, GSM, Royal College of Surgeons in Ireland, Dublin, Leinster, Ireland

Thank you for your suggestion – we have identified the reviewers throughout the screening section by adding their initials.
